# Genomewide Analysis of Aryl Hydrocarbon Receptor Binding Targets Reveals an Extensive Array of Gene Clusters that Control Morphogenetic and Developmental Programs

**DOI:** 10.1289/ehp.0800485

**Published:** 2009-03-24

**Authors:** Maureen A. Sartor, Michael Schnekenburger, Jennifer L. Marlowe, John F. Reichard, Ying Wang, Yunxia Fan, Ci Ma, Saikumar Karyala, Danielle Halbleib, Xiangdong Liu, Mario Medvedovic, Alvaro Puga

**Affiliations:** 1 Laboratory for Statistical Genomics and Systems Biology and; 2 Division of Environmental Genetics and Molecular Toxicology, Department of Environmental Health and Center for Environmental Genetics, University of Cincinnati College of Medicine, Cincinnati, Ohio, USA

**Keywords:** Ah receptor, ChIP-on-chip, gene-environment interactions, gene regulation, gene target networks

## Abstract

**Background:**

The vertebrate aryl hydrocarbon receptor (AHR) is a ligand-activated transcription factor that regulates cellular responses to environmental polycyclic and halogenated compounds. The naive receptor is believed to reside in an inactive cytosolic complex that translocates to the nucleus and induces transcription of xenobiotic detoxification genes after activation by ligand.

**Objectives:**

We conducted an integrative genomewide analysis of AHR gene targets in mouse hepatoma cells and determined whether AHR regulatory functions may take place in the absence of an exogenous ligand.

**Methods:**

The network of AHR-binding targets in the mouse genome was mapped through a multipronged approach involving chromatin immunoprecipitation/chip and global gene expression signatures. The findings were integrated into a prior functional knowledge base from Gene Ontology, interaction networks, Kyoto Encyclopedia of Genes and Genomes pathways, sequence motif analysis, and literature molecular concepts.

**Results:**

We found the naive receptor in unstimulated cells bound to an extensive array of gene clusters with functions in regulation of gene expression, differentiation, and pattern specification, connecting multiple morphogenetic and developmental programs. Activation by the ligand displaced the receptor from some of these targets toward sites in the promoters of xenobiotic metabolism genes.

**Conclusions:**

The vertebrate AHR appears to possess unsuspected regulatory functions that may be potential targets of environmental injury.

The aryl hydrocarbon receptor (AHR) is a cytosolic ligand-activated transcription factor in the basic region–helix-loop-helix–PER-ARNT-SIM (bHLH-PAS) family of proteins (Crews and Fan 1999). Its activation by prototypical ligands such as benzo[*a*]pyrene (BaP), 2,3,7,8-tetrachlorodibenzo-*p*-dioxin (TCDD), coplanar polychlorinated biphenyls, and many other ligands causes its translocation to the nucleus, where it dimerizes with a second bHLH-PAS protein, aryl hydrocarbon receptor nuclear translocator (ARNT), to form a heterodimeric transcription factor. AHR/ARNT complexes bind to canonical DNA consensus sequences and initiate transcription of genes coding for many phase I and phase II detoxification enzymes (Hankinson 1995). In addition, the AHR complex regulates the expression of genes whose products control a broad spectrum of cellular functions, such as cell division and cell fate (Marlowe and Puga 2005). Because so many of its ligands are known or suspected carcinogens, a disproportionate amount of AHR research has focused on the analysis of the toxicologic or adaptive end points that it mediates. Over the years, however, the AHR has also been found to activate signal transduction pathways that run parallel to these detoxification pathways, often in the absence of stimulation by an exogenous ligand (Marlowe and Puga 2005).

Mice with a homozygous ablation of the *Ahr* gene are viable but suffer numerous age-related pathologies involving multiple organ systems (Fernandez-Salguero et al. 1995), suggesting that the AHR has endogenous functions that do not require activation by xenobiotic ligands and that play an important role in maintenance of cellular homeostasis (Bock and Kohle 2006). Teratogenic end points of dioxin, such as the induction of cleft palate in mouse embryos (Abbott et al. 1994), are at the crossroads of developmental and toxic signals, providing important clues to the nature of such homeostatic AHR functions. Receptor activation by environmental chemicals is likely to have the dual effect of disrupting homeostasis while simultaneously triggering the induction of detoxification pathways. Testing this possibility is one of the main objectives of the present study.

The ancestral function of the AHR appears to be the regulation of specific aspects of embryonic development, having acquired the ability to bind xenobiotic compounds only during vertebrate evolution (Hahn 2002). The invertebrate AHR protein also functions as a transcription factor and binds to the same dimerization partner and *cis*-acting response elements as the vertebrate protein, but it does not respond to any of the environmental ligands recognized by the vertebrate receptor. Instead, it regulates diverse developmental processes that are independent of toxicant or of exogenous ligand exposure, such as neuronal differentiation during development in *Caenorhabditis elegans* (Qin and Powell-Coffman 2004) and normal morphogenesis of legs, antennae, and bristles in *Drosophila melanogaster* (Adachi-Yamada et al. 2005). In keeping with this role in invertebrates, the mammalian AHR also possesses a developmental role in craniofacial, renal, and cardiovascular morphogenesis (Birnbaum et al. 1989; Fernandez-Salguero et al. 1997; Lahvis et al. 2005), raising the key question of whether its ancestral function has been retained during vertebrate evolution.

Knowledge of the range of genes directly regulated by the AHR would facilitate integration at a systems biology level of the complex physiologic mechanisms that this receptor regulates both endogenously and in response to toxic ligands. In the present study, we combined multiple technologies and knowledge bases to conduct an integrative genomewide analysis of AHR gene targets in mouse hepatoma cells. Assuming no *a priori* knowledge of the results of the present study, we conclude that the AHR is likely a key regulator of genes with a function during embryonic development, suggesting that gene–environment interactions during fetal life may be potential triggers of developmental abnormalities.

## Materials and Methods

A detailed description of all materials and methods used can be found in the Supplemental Material (available online at http://www.ehponline.org/members/0800485/suppl.pdf).

### Cell culture and chemical treatments

Mouse Hepa-1c1c7 (Hepa-1) cells, and its mutant derivative *c*35 expressing a DNA-binding defective AHR (Sun et al. 1997), were grown in α-minimal essential medium supplemented with 5% (vol/vol) fetal bovine serum, 26 mM NaHCO_3_, and 1% (vol/vol) antibiotic-antimycotic mixture at 37°C in a humidified 5% CO_2_ atmosphere. When the cells reached 70–80% confluence, we treated them with the standard ligand concentrations that give maximal induction responses for xenobiotic detoxification genes, 5 μM BaP or 5 nM TCDD, and 0.1% dimethyl sulfoxide (DMSO) vehicle as control. Cells were then harvested 1.5 hr and 8 hr posttreatment for chromatin immunoprecipitation (ChIP) and RNA extraction, respectively.

### Transcriptome analysis: experimental design and procedure

We performed comparative transcriptome profiling of DMSO-, BaP-, and TCDD-treated Hepa-1c1c7 and *c*35 cells using the Affymetrix GeneChip Mouse Genome 430 2.0 Array (Santa Clara, CA). Eight hours posttreatment, three individual RNA extractions from three biological replicates of 5 × 10^6^ cells were prepared, and total RNA was extracted using NucleoSpin RNA II columns (Macherey-Nagel; Bethlehem, PA). We validated RNA extracts for quality and integrity using the Agilent 2100 Bioanalyzer (Santa Clara, CA).

### ChIP and target preparation

We performed ChIP as previously described (Schnekenburger et al. 2007), using ChIP-verified anti-AHR antibody (Biomol, Plymouth Meeting, PA) and control nonimmune rabbit IgG (Upstate, Billerica, MA). An aliquot of immunoprecipitated purified DNA was checked for immunoprecipitation efficiency by real-time polymerase chain reaction (PCR) using primers specific for the mouse *Cyp1a1* promoter domains known to be bound by AHR. We observed a significant enrichment (16- to 32-fold) for the specific AHR target relative to the control antibody. For target preparation, immunoprecipitated DNA was PCR-amplified and labeled according to the standard protocol supplied by Affymetrix (http://www.affymetrix.com/products/arrays/specific/mouse_promoter.affx).

### Quantitative real-time PCR analysis

We performed quantitative polymerase chain reactions (QPCRs) in triplicate for approximately 6% of the 750 top-ranked genes scored by whole-genome hybridization and established the statistical significance of the differential expression by the empirical Bayes *t*-test as implemented in the Bioconductor limma package (Smyth 2004; http://bioinf.wehi.edu.au/limma/). *p*-Values were adjusted for multiple comparisons using Benjamini-Hochberg false discovery rate (FDR) procedures (Benjamini and Hochberg 1995).

### Expression microarray hybridization and analysis

Hybridization to Affymetrix GeneChip Mouse Genome Arrays was performed with biotinylated amplified total RNA, followed by staining and washing using the Affymetrix GeneChip Hybridization Wash and Stain Kit. Analysis of differentially expressed genes used R statistical software (R Project 2009) and the Bioconductor limma package (Smyth 2004). We assessed chip quality using the affyQCReport package of Bioconductor. Estimated fold changes for each comparison were calculated using analysis of variance (ANOVA) and significance was assessed using an intensity-based Bayesian moderated *t*-statistic (IBMT) (Sartor et al. 2006). We considered genes satisfying an FDR < 0.05 level of significance and showing a minimum fold change > 50% to be differentially expressed.

### Binding site identification

Raw data were quantile normalized and all array-pairwise Pearson correlation coefficients were determined to be > 0.95. We used the Bioconductor limma package (Smyth 2004) to perform ANOVA on normalized probes and calculated significance levels for probes using IBMT (Sartor et al. 2006). We removed from subsequent analyses probes with intensities in the lowest 20% [see Supplemental Material (http://www.ehponline.org/members/0800485/suppl.pdf)] for the identification of AHR-bound gene promoters.

### Functional analysis and clustering

Gene Ontology (GO) (2009) terms and Kyoto Encyclopedia of Genes and Genomes (KEGG) (2009) pathways were tested for enrichment of binding and/or differential expression using Fisher’s exact test. For ChIP/chip conditions, we used the list of 750 top-ranked genes; for the 5-nucleotide motif, we identified 562 genes with ≥ 12 sites within 2 kb of the transcription start site (TSS); and for the longer motif, we identified 693 genes with at least two sites in the promoter and at least one of those within 2 kb of the start site. For expression results, we also used the 750 top-ranked up-/down-regulated genes in wild-type versus *c*35 cells. These gene lists were then clustered based on their level of enrichment in the biological processes in GO [see Supplemental Material (http://www.ehponline.org/members/0800485/suppl.pdf) for further details of the methods. Clustering can be viewed interactively at http://eh3.uc.edu/supplements/ahrchip by following the link Hierarchical clustering of enriched GO terms at the bottom of the page].

## Results

### Strategy for AHR target gene network mapping

**Figure 1:**
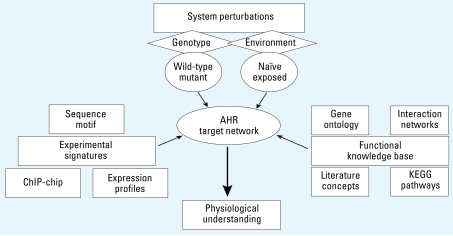
Schematic representation of the integrative approach. To develop a comprehensive view of the AHR target network, we analyzed gene expression and DNA binding data under a variety of gene–environment combinations, including wild-type cells and cells with a DNA-binding–deficient mutant AHR, in naive cells and in cells exposed to two different ligands, BaP and TCDD. Experimental signatures under these conditions were determined by ChIP/chip assays, binding sequence motif analysis, and global gene expression profiling. Data were combined with an extensive prior functional knowledge base derived from GO, interaction networks, KEGG pathways, and literature molecular concepts. Ultimately, this integrated approach will lead to a physiologic understanding of indigenous AHR functions and of the molecular basis of gene–environment interactions controlled by the AHR that lead to the derailment of normal biological processes.

Evidence accumulated over the years shows that the network of genes regulated by the vertebrate AHR may extend well beyond those genes induced as a consequence of exposure to xenobiotic ligands (Barouki et al. 2007). To develop a comprehensive analysis of genomic sites bound by the AHR and of possible target changes resulting from perturbation of gene–environment cues, we used mouse hepatoma Hepa-1c1c7 cells, which have been extensively studied in AHR signaling work, exposed to either of two AHR ligands, BaP or TCDD, or in an exogenous ligand-naive environment exposed to the control solvent DMSO. To provide direct verification of the unique and specific role of the AHR protein in the results, we compared binding data from these cells with data from the Hepa-1c1c7 mutant derivative *c*35 cell line, which has a missense mutation in the *Ahr* gene. This mutation blocks the ability of the AHR protein to bind DNA, although the mutated AHR protein retains its ability to bind ligand and translocate to the nucleus (Sun et al. 1997). Under these conditions, we identified the AHR network of target binding sites with a multipronged approach that included the collection of experimental signatures and their integration into a prior functional knowledge base. ChIP with anti-AHR or nonimmune IgG antibodies was followed by whole-genome amplification and microarray hybridization (ChIP/chip) to the Affymetrix GeneChip Mouse Promoter 1.0R Array. ChIP/chip information, binding sequence motif analysis, and global gene expression profiling, using the Affymetrix GeneChip Mouse Genome 430 2.0 Array, provided one arm of the approach, which we combined with an extensive prior functional knowledge derived from GO, interaction networks, KEGG pathways, and literature molecular concepts. Figure 1 shows a schematic representation of the integrative approach, focused on developing a comprehensive physiologic understanding of this gene’s regulatory network.

### AHR ChIP/chip profiling

ChIP/chip experiments showed distinct AHR binding signals in naive and ligand-exposed wild-type Hepa-1c1c7 cells but no signal in the mutant *c*35 cells, indicating that the signal was entirely AHR dependent. The probe density in the array chip was biased in the neighborhood of the TSS (± 1 kb), the same region where the highest binding signal intensity for the top 15% of probes was also strongest [see Supplemental Material, Figure 1A (http://www.ehponline.org/docs/2009/0800485/suppl.pdf)]. After normalizing for probe density distribution, we found AHR-bound regions encompassing multiple consecutive probes to be preferentially located in the neighborhood of the TSS for naive as well as ligand-exposed wild-type cells, although we also identified many such regions outside this 2-kb symmetric region around the TSS [see Supplemental Material, Figure 1B (http://www.ehponline.org/docs/2009/0800485/suppl.pdf)]. Surprisingly, the strongest signal within 1 kb of the TSS was not in ligand-exposed cells, but in naive DMSO-treated cells. In contrast, *c*35 cells, whether naive or TCDD treated, showed no evidence of preferential binding along gene promoters, indicative of the inability of the mutant AHR in these cells to bind to DNA [see Supplemental Material, Figure 1B (http://www.ehponline.org/docs/2009/0800485/suppl.pdf)].

To identify the most likely AHR targets under each experimental condition, we selected genes based on the statistical significance of their overall *z*-score of binding. This resulted in approximately 750 genes significantly bound by the AHR in naive, DMSO-treated wild-type cells, using a local FDR < 0.10 criterion (Efron 2004; Taylor et al. 2005). All three comparisons of anti-AHR versus IgG immunoprecipitations from DMSO-, TCDD-, and BaP-treated cells resulted in approximately 750 ± 30 unique genes satisfying the criterion of *z*-score > 4.0 (*p* < 1.6 × 10^−5^). Supplemental Material, Table 1 (http://www.ehponline.org/docs/2009/0800485/suppl.pdf) lists the 750 top-ranked genes for each experimental comparison. Three unexpected findings clearly stand out from these initial analysis results. First is the large number of gene promoter regions that show significant AHR binding in naive cells, in agreement with the high signal observed around the TSS and the high proportion of sites to satisfy the specified binding criteria in naive cells [see Supplemental Material (http://www.ehponline.org/docs/2009/0800485/suppl.pdf) for the derivation of parameter *p_c_* under “Gene scoring algorithm”]. This finding is suggestive of a physiologic role for the receptor in the absence of an exogenous ligand. Second is the large proportion of top-ranked genes in naive cells that have functions involved in transcriptional regulation, including a large number of homeobox genes. Third is the large fraction of top-ranked AHR-binding genes in naive cells that are not found in ligand-treated cells and the comparative gain of other genes in cells exposed to either ligand. Supplemental Material, Figure 2 (http://www.ehponline.org/docs/2009/0800485/suppl.pdf) shows differential binding profiles for a select group of genes chosen from among the 750 top-ranked genes.

**Figure 2:**
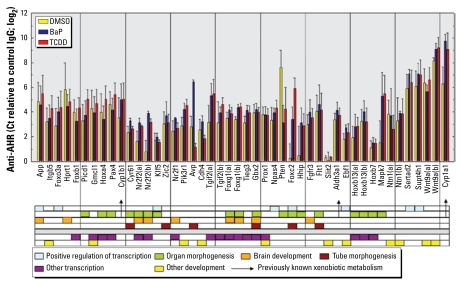
Independent verification of ChIP/chip results by real-time QPCR of a select group of genes. Primer pairs were selected to amplify short segments (< 200 bp) of the promoter regions of the genes shown here. The *y*-axis shows the log_2_ ratio of the QPCR C_t_ values of amplified DNA from ChIP with anti-AHR antibodies relative to nonimmune control IgG.

To independently validate the ChIP/chip results, a second set of ChIP experiments was performed on chromatin extracts from naive- and BaP- and TCDD-treated cells by two researchers not involved in the first experimental series. We processed these samples by real-time QPCR for the detection of 46 promoter regions chosen from 41 unique genes among the 750 top-ranked genes [see Supplemental Material, Table 1 (http://www.ehponline.org/docs/2009/0800485/suppl.pdf)], plus *Cyp1a1* added as a positive control. These genes were not selected at random, but were chosen on the basis of a *z*-score > 4, and their membership in a significantly enriched GO term or KEGG pathway. They belong to widely differing functional categories, including regulation of transcription, organ morphogenesis, brain development, and others not expected from prior knowledge to be regulated by the AHR. Also included were xenobiotic metabolism genes, known to be regulated by the AHR, and several tumor suppressor and other genes involved in the onset or progression of proliferative diseases, such as *Pten*, *Foxo3a*, *Cyr61*, *Prox1*, and *Pik3r1*. [see Supplemental Material, Table 1 (http://www.ehponline.org/members/0800485/suppl.pdf) for the Entrez identification and accession number of all genes mentioned in this article]. *Pten* was one of the genes showing higher AHR binding in naive cells than in BaP- or TCDD-treated cells, a finding that we verified by the validation experiments (*p* = 0.0025 for BaP and *p* = 0.0155 for TCDD). All but four of the 46 promoter regions were verified (FDR < 0.10) for at least one of either the naive (39 of 46) or BaP- (42 of 46) or TCDD- (40 or 46) treated cells (Figure 2).

### Functional analysis of AHR bound genes in naive and in ligand-activated cells

We performed functional enrichment analysis with the 750 top-ranked ChIP/chip genes of all comparisons using KEGG pathways, GO, and Ingenuity pathway analysis (http://www.ingenuity.com) The top enriched biological processes for naive cells included anatomical structure morphogenesis; organ morphogenesis; blood vessel morphogenesis; vasculature development; positive regulation of nucleobase; nucleoside, nucleotide, and nucleic acid metabolic processes; angiogenesis; positive regulation of cellular metabolic processes; blood vessel development; and embryonic morphogenesis (*p* < 0.001; FDR < 0.15). At this level of significance, KEGG did not reveal any enriched pathways in naive cells. The top-scoring Ingenuity pathway network for naive cells (score = 50) included the transcription factors cJUN and NR2F1 [Supplemental Material, Figure 3 (http://www.ehponline.org/docs/2009/0800485/suppl.pdf)], both of which have previously been shown to interact with the AHR (Hoffer et al. 1996; Klinge et al. 2000) and the WNT pathway, which is also known to interact with the AHR (Mathew et al. 2008). In agreement with our findings using GO categories, this Ingenuity pathway network was significantly enriched with the function development of organs (*p* = 1.08 × 10^−8^) and development of brain (*p* = 1.48 × 10^−6^). As the negative control, we tested the top 750 genes from the ChIP of naive *c*35 cells for enriched functional categories in GO and found no significantly enriched categories (all *q*-values = 1).

The functional enrichment analysis of the genes determined to be more highly bound by AHR when cells were treated with TCDD or BaP than when they were in the naive state identified the KEGG pathway “metabolism of xenobiotics by cyto-chrome P450” as enriched when cells were exposed to either ligand (BaP, *p* = 0.00056; TCDD, *p* = 0.039). Specific genes included *Aldh3a1* (both ligands), *Cyp1b1* (TCDD), *Gstk1* (BaP), *Gstt1* (TCDD), *Ephx1* (BaP), *Cyp3a41a* (BaP), *Cyp3a16* (both ligands), *Cyp2c55* (TCDD), *Gsto2* (BaP), *Ugt1a10* (BaP), *Ugt1a6a* (BaP), *Ugt1a2* (BaP), *Gstm1* (BaP), *Cyp2f2* (TCDD), and *Mgst1* (TCDD). Previously unbeknownst to us, the promoter of the best known of the positive controls, *Cyp1a1*, was incorrectly tiled on the mouse Affymetrix promoter array and therefore was absent from the ChIP-chip analyses.

### Integration of sequence signatures into the AHR target network

**Figure 3:**
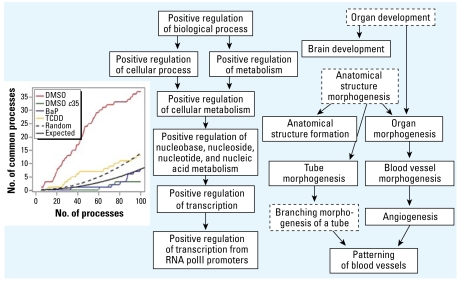
Indigenous AHR functions in naive cells. Functional categories identified based on ChIP/chip results from naive cells were integrated with those from sequence data, resulting in 14 overlapping terms with *p* < 0.01, significantly more than expected by chance (maximum of three overlapping from 1,000 random simulations; *p* << 0.001). These overlapping categories delineated the two process lineages shown, one relating to positive regulation of biological processes and leading to the regulation of transcription, and the other including many developmental processes. The three processes framed with dashed lines were significant at *p* < 0.05 and are included here for continuity. The inset shows the number of GO terms in common between various enrichment tests and enrichments based on the five-nucleotide canonical motif. Ranked lists of GO terms were calculated for ChIP/chip data of naive wild-type, and BaP- and TCDD-treated cells, naive *c*35 cells, and random simulations. The inset plots the number of overlapping terms between each set and enrichments based on the five-nucleotide canonical motif for increasing length of ranked lists, as well as the expected values based on Fisher’s exact test.

To assess whether the genes detected by AHR ChIP/chip conformed to the presence of previously established AHR binding sites, we analyzed two AHR binding motifs, the canonical 5-nucleotide motif 5′-GCGTG-3′ and the longer 18-bp motif 5′-CCYCNRRSTNGCGTGASA-3′ derived from nine binding sites in the mouse and rat P450 genes, as defined by the TRANSFAC position weight matrix (PWM) V$AHR_01 (Matys et al. 2003). Both motifs showed the same distribution of relative positions with respect to the TSS of known genes, but of the two motifs, the shorter [see Supplemental Material, Figure 4A (http://www.ehponline.org/docs/2009/0800485/suppl.pdf)] gave a higher peak signal-to-noise ratio than did the longer motif (4.5- vs. 3-fold ratio) [see Supplemental Material, Figure 4B (http://www.ehponline.org/docs/2009/0800485/suppl.pdf)]. Functional enrichment analyses comparing the categories of genes significantly enriched in the canonical binding motif with categories identified on the basis of ChIP/chip results from naive cells (see “Materials and Methods”) revealed 14 biological processes that were enriched in both analyses at the *p* < 0.01 level. Based on our random simulations accounting for the correlations in the GO hierarchical structure, such a high number of processes in common with *p* < 0.01 was highly unlikely to have occurred by chance (*p* << 0.001; maximum of three overlapping from 1,000 random simulations). These overlapping categories delineated two process lineages, one relating to regulation of transcription and the other including several developmental processes, such as brain development, blood vessel morphogenesis (with its parent, organ morphogenesis), and tube morphogenesis (Figure 3). The inset in Figure 3 illustrates the degree of overlap in functional enrichment results between sequence motif and several ChIP/chip comparisons. We ranked biological processes in terms of significance for all functional enrichment tests and calculated the numbers of processes in common for increasing length of ranked lists for results based on sequence motif versus ChIP/chip results from naive, BaP-treated, TCDD-treated, and negative control *c*35 cells. To determine the number of processes in common that could be expected to be enriched by chance, we calculated the mean number of overlapping terms from 1,000 random simulations between sequence motif and naive wild-type cells (dashed line) as well as the expected overlap by chance based on Fisher’s exact test (solid black line), again for increasing length of ranked lists. Thus, the inset to Figure 3 shows the degree of overlap in enriched biological processes for the entire range of the top 5–100 ranked processes, rather than for any single cutoff alone. Using the naive *c*35 cells as a negative control, we found no significant overlap between top-ranked biological processes in these cells and those identified on the basis of sequence motif knowledge. These results support and extend the findings based on ChIP/chip data alone.

### Distribution of AHR binding sites in genes predicted by ChIP/chip

To characterize the distribution of predicted AHR binding sites in the promoters of genes belonging to the enriched processes found in the functional analyses, we created histograms of relative locations of predicted AHR motif locations for genes identified by ChIP/chip in enriched categories, and for genes in the same enriched categories that were not identified by ChIP/chip. Specific enriched categories used were brain development, blood vessel morphogenesis, tube morphogenesis, positive regulation of transcription, anatomical structure formation, and, to serve as a negative control, membrane. For each gene list, we also created a histogram of the overall background distribution of predicted AHR motifs around known TSS. Enrichment of AHR motifs was evident in those genes that scored by ChIP/chip [see Supplemental Material, Figure 4A (http://www.ehponline.org/docs/2009/0800485/suppl.pdf) compared with the genes in the same categories that did not score [see Supplemental Material, Figure 4B (http://www.ehponline.org/docs/2009/0800485/suppl.pdf)]. When we based the analysis on top-ranked ChIP/chip genes assigned to any enriched biological process on the basis of ChIP/chip data alone (i.e., not using any sequence-based information), the pattern of genes identified by ChIP/chip was again distinguishable from the pattern of genes not identified by ChIP/chip, which was closer to the pattern observed for all genes [see Supplemental Material, Figure 4C (http://www.ehponline.org/docs/2009/0800485/suppl.pdf)]. Although a quantitative estimate of power is not feasible for the ChIP/chip assay, these graphs provide strong evidence that our experiments identified a high percentage of the AHR target genes involved in these biological processes.

**Figure 4:**
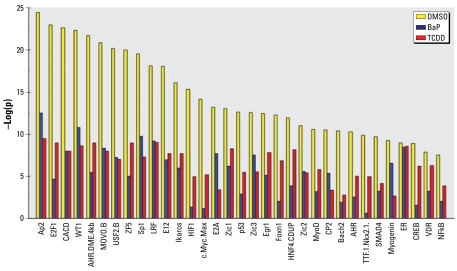
Transcription factor overlap with the AHR network. For each of the 267 mouse transcription factors with at least one PWM in the TRANSFAC version 12.1 database and two novel entries we developed for AHR, we scored genes as to how likely they were to have a binding motif within 2 kb at either side of their TSS. For each transcription factor, we used the nonparametric Mann–Whitney ranking test and Fisher’s exact test (using a top 10th percentile cutoff for motifs) to calculate whether the top-scoring ChIP/chip genes had significantly more binding motifs than the rest of the genes. The graph shows the results of the Mann–Whitney ranking test for the top 30 transcription factors mapping to the same promoter regions as the AHR.

Rarely is a gene regulated by a single transcription factor binding in isolation. More often, promoters of developmentally regulated and other genes have binding sites for multiple transcription factors that bind either simultaneously in a complex, or sequentially, as developmental or metabolic demands so require. To identify transcription factors that potentially interact with the AHR or work to coregulate genes with AHR, we examined the overlap between genes predicted to have a specific transcription factor binding motif in their promoters and genes whose promoters were bound by AHR, as determined from our ChIP/chip results. For each of the 267 mouse transcription factors with at least one PWM in the TRANSFAC version 12.1 database, we scored genes based on to how likely they were to have such a motif within 2 kb at either side of their TSS. For each transcription factor, we then calculated whether the top-scoring ChIP/chip genes had significantly more binding motifs than the rest of the genes using the nonparametric Mann–Whitney ranking test and Fisher’s exact test for the top 10% highest-scoring genes (see “Materials and Methods”). The two tests were in close agreement (Pearson’s correlation between *p*-values, *r* > 0.82 for all three experimental conditions). The results with *c*35 mutant cells served as a negative control, and indeed, they showed no significant interacting transcription factors at the FDR < 0.10 level. We identified numerous transcription factors having an odds ratio of enrichment > 1.5 and FDR < 0.01 for both statistical tests. These included general transcription factors involved in developmental processes (e.g., USF and E2A), proteins coded for by homeobox genes (e.g., *Foxn1*, *Pax5*, and *Nkx5.1*), and several other transcription factors [estrogen receptor (ER), HIF, Myc/Max, CREB, p53, and HNF4] previously known or suspected to interact with the AHR (Barouki et al. 2007). Combinatorial regulatory interactions between ER and AHR are the subject of much current investigation (Pocar et al. 2005). Although we detected similar interacting transcription factors with the genes in all three experimental settings, the genes bound by AHR in the naive state provided a stronger enrichment of interacting transcription factors in general and of the AHR in particular. Overall, 70, 9, and 20 transcription factors motifs were found for DMSO-, BaP-, and TCDD-treated cells, respectively, at the FDR < 0.01 level in both the Fisher’s exact test and Mann–Whitney test. Figure 4 shows significance values [as − log(*p*)] of the Mann–Whitney test for a select set of transcription factor TRANSFAC entries for ChIP results from naive and BaP- and TCDD-treated cells.

### Generation of an alternative positional weight matrix (PWM) for the AHR

We tested the individual AHR PWMs defined by TRANSFAC separately, to determine whether the promoters of the top-ranked ChIP/chip genes had greater enrichment with one than with the other. Of the two individual AHR PWMs defined by TRANSFAC, V$AHR_ Q5 (M00778) scored very high (Fisher’s exact *p* = 7.5 × 10^−8^, 0.0021, and 0.00019 for DMSO, BaP, and TCDD, respectively), whereas the other, V$AHR_01 (M00139), was not significant (*p* = 0.060, 0.13, and 0.16). This result suggests that PWM V$AHR_Q5 (M00778), which is virtually identical to the canonical five-nucleotide motif 5′-GCGTG-3′, may provide the more accurate description of the murine AHR binding motif, given the present data.

Bound promoter sequences of the QPCR-verified genes were used to identify an alternative PWM for the AHR. Fragments of 100 bp, each including the short five-nucleotide motif (5′-GCGTG-3′), were input into Discriminating Matrix Enumerator (DME) software (Smith et al. 2005). We obtained most discriminating six-, seven-, eight-, and nine-nucleotide PWMs using two different random promoter regions as background, one 2 kb on either side of TSS (AHR. DME.4kb), and the other 7 kb upstream to 3 kb downstream of TSS (AHR.DME.10kb) [see Supplemental Material, Table 2 (http://www.ehponline.org/docs/2009/0800485/suppl.pdf)]. By removing the genes whose promoter sequences were used in creating these new PWMs, we were able to perform an unbiased comparison of the new PWMs and the previously defined TRANSFAC PWMs for AHR. Using the rest of the ChIP/chip identified genes for DMSO-, BaP-, and TCDD-treated cells and the same methods as described above, we tested for enrichment of these motifs. The results in Fisher’s exact test showed that the previously defined AHR in TRANSFAC was slightly more highly significant, whereas Mann–Whitney *U*-tests resulted in our novel motifs being more significant [see Supplemental Material, Table 2 (http://www.ehponline.org/docs/2009/0800485/suppl.pdf)]. We provide here our novel AHR PWMs as an alternative definition of equal or perhaps greater quality than that previously defined. Figure 4 includes two additional entries for the AHR constructed based on our experimental results using the DME software (Smith et al. 2005). Using the same approach, we tested the individual AHR PWMs defined by TRANSFAC separately and identified novel PWMs using ChIP regions verified by real-time QPCR.

### Integration of expression profiling into the AHR target network

The AHR transcriptional induction profile has been extensively studied, whether activated by TCDD, by BaP, or in the absence of exogenous ligands (Frericks et al. 2007). In addition to using prior knowledge to integrate expression profiles into the AHR gene target network, we performed a new set of expression profile analyses of the wild-type Hepa-1c1c7 and the mutant *c*35 cell lines and compared the responses in naive cells with responses in cells exposed to TCDD or BaP for 8 hr. Results of our new expression array studies were in close agreement with current knowledge. For example, present among the 74 genes significant at FDR < 0.05 level were *Cyp1a1*, *Aldh3a1*, *Nqo1*, *Adh7*, *Cyp1b1*, *Hspa4l*, *Vegfc*, *Xdh*, *Tiparp*, and *Tnfaip2*. Functional enrichment analysis identified metabolism of xenobiotics by cytochrome P450 as the sole enriched category or pathway (FDR < 0.1) involving the genes *Adh7*, *Cyp1a1*, *Cyp1b1*, *Aldh3a1*, and *Cyp2s1* in BaP- and TCDD-treated cells; additionally, *Adh1* was induced by TCDD only and *Mgst2* by BaP only. The difference in response to ligand between wild-type and *c*35 cells was stark. No single, individual gene was significantly differentially expressed at the FDR < 0.1 level for the mutant *c*35 line under TCDD or BaP treatment.

The differential expression profile between naive wild-type and mutant cells involved several thousand genes. Functional analysis of genes more highly expressed in wild-type cells identified many of the same processes already found on the basis of overlapping ChIP/chip and sequence data. Specifically, at the *p* < 0.01 level, overlapping processes included positive regulation of cellular processes, positive regulation of transcription, blood vessel development, vasculature development, and organ morphogenesis. At the *p* < 0.05 cutoff level, we additionally identified terms related to cell cycle arrest and regulation of growth as being enriched in both ChIP/chip and expression data, in good agreement with known differences previously observed between *Ahr*^+/+^ and *Ahr*^−/−^ cells (Chang et al. 2007). Genes in some other groups such as brain development that we found to be enriched by ChIP/chip and sequence data were not significantly more highly expressed in wild-type than in *c*35 cells. This may be the consequence of these genes not being expressed in hepatoma cells, but yet having a poised AHR that may still occupy the promoter at appropriate motif sites without causing changes in transcription.

### Clustering of enriched processes for an overall integrative view

**Figure 5:**
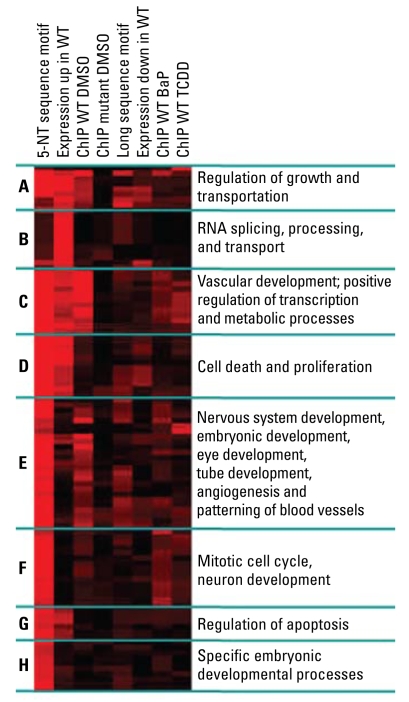
Functional heat map of GO biological processes that are AHR targets. Abbreviations: 5-NT, five nucleotide; WT, wild type. The clustering of GO biological process enrichment scores visually integrates three independent data sources—ChIP/chip, sequence motif, and expression profiles. The clustering is divided into eight distinct clusters, labeled A–H, each contributing significant information to the overall interpretation of results. Each column represents enrichment results of one individual comparison from one of the three data sources. Eighty categories were first identified containing > 10 and < 1,000 genes that were enriched within at least one of the gene lists (FDR < 0.2). For each gene list, an “enrichment profile” was constructed consisting of –log(Fisher’s test *p*-value of enrichment) for each of these 80 GO terms. Gene lists and GO terms were then clustered based on such “enrichment profiles” using the simple Euclidian-distance–based average-linkage hierarchical clustering.

Integration of ChIP/chip information, sequence motif data, and expression analysis with prior functional knowledge converged on the overall clustering heat map of GO biological processes identified as enriched in one or more experimental conditions (Figure 5). The clustering of functional enrichment significance scores visually integrates three independent data sources—ChIP/chip, sequence motif, and expression profiles. We divided the clustering into eight distinct clusters of categories, labeled A–H, each contributing significant information to the overall interpretation of results. Note that, as would be expected, virtually all clusters are identified based on sequence motif information, and that no cluster is enriched in the ChIP/chip data of naive *c*35 mutant cells. Cluster A comprises processes identified by sequence, by ChIP/chip in naive wild-type cells, and as up-regulated in wild-type versus *c*35 mutant, but not by ChIP/chip in BaP- or TCDD-treated wild-type cells. These mainly represent processes involved in regulation of growth and transcription. Cluster C also represents processes identified by sequence and ChIP/chip in naive wild-type cells and by up-regulation in expression of wild-type versus c35 mutant cells, and to a certain extent in BaP- and TCDD-treated wild-type cells. These include positive regulation of transcription, vascular development, organ morphogenesis, and metabolic processes. Cluster B contains processes enriched by genes with increased expression in wild-type versus c35 cells, including processes related to mRNA processing and transport, suggestive of the likelihood that these processes are independent of AHR binding to DNA. Many of these genes do not have canonical AHR binding sites, which raises the possibility that AHR binds to them indirectly, through protein–protein interactions with other transcription factors, as is the case of AHR–retinoblastoma (RB) and AHR–E2F1 interactions (Ge and Elferink 1998; Marlowe et al. 2008). Clusters D and G comprise processes related to cell death, proliferation, and regulation of apoptosis and are enriched based on sequence and expression data, but not ChIP/chip, of naive cells. These might be involved in regulatory mechanisms that include cyclical, short-lived recruitment of the AHR to gene promoters, such as has previously been observed for other AHR ligands (Hestermann and Brown 2003). Cluster E represents gene groups identified by sequence and ChIP/chip data and comprises several processes related to nervous system development, embryonic development, eye development, tube morphogenesis, angiogenesis, and patterning of blood vessels. Most of these were not well enriched in expression data, and thus they may comprise sets of genes on whose promoters AHR is poised but not currently active or that may be silenced by epigenetic mechanisms, such as hypermethylation. Cluster F consists of processes enriched mainly based on sequence and, to a certain extent, ChIP/chip of naive and/or BaP- and TCDD-treated cells, and includes relatives of mitotic cell cycle and neuron development. Those processes enriched in this cluster may be representative of AHR-dependent, xenobiotic-induced gene regulation. We identified the processes in cluster H based on sequence alone and included processes related to specific embryonic and other developmental processes, such as chordate embryonic development, regulation of synaptic plasticity, auditory receptor cell differentiation, and embryonic development ending in birth or egg hatching. The genes involved in these processes may not be susceptible of activation in hepatoma cells.

## Discussion

In this study, we have identified a genome-wide network of AHR-regulated target genes and their biological functions by integrating information from our analyses of ChIP, binding motifs, and global gene expression. These analyses have been conducted with a mouse hepatoma cell line commonly used in AHR signaling work and have been controlled at every step by similar studies done on a derivative cell line bearing a receptor with a missense mutation that inhibits its binding to DNA, hence providing an important biological verification of the results. The network so identified constitutes a critical new resource for the study of AHR-regulated gene expression that will help generate a better understanding of the physiologic role of the AHR in the intact organism and of the health effects of human population exposure to AHR ligands.

Some of our findings are indeed surprising, and it could be argued that they are spurious, because of contaminating cross-reactivities in our anti-AHR and control IgG antibodies. This and other trivial explanations for our results are ruled out by the control experiments with *c*35 cells, which, should the purity of our antibodies be questionable, would have given similar results as the experimental Hepa-1 cells. Although the current paradigm of AHR function involves activation by xenobiotic ligands as an obligatory step in AHR translocation and function, our data show that the receptor not only occupies a significant number of its target promoters in naive, unstimulated cells, but also actively participates in the regulation of many of the genes that it occupies under these conditions. However surprising in their extent and magnitude, these results are not totally unexpected and to some extent could have been predicted, given the abundant examples in the literature of a regulatory role for the AHR in the absence of xenobiotic ligand activation both in cultured cells and *in vivo*. By mechanisms independent of xenobiotic metabolism, AHR knockout mice suffer from an impaired cardiovascular phenotype, which includes the failure of fetal vascular structures in the liver and eye to undergo apoptosis (Lahvis et al. 2000), and from a fibrotic hepatic phenotype, complicated with premature senescence characteristics (Fernandez-Salguero et al. 1995). In addition, a growing body of experimental evidence strongly suggests a role for the AHR in cell proliferation, differentiation, and liver and immune system homeostasis, as well as in tumor development (Barouki et al. 2007). Moreover, failure of specific GABAergic neurons to develop is the hallmark of ablation of the *C. elegans Ahr* ortholog (Qin and Powell-Coffman 2004), as is the abnormal morphogenesis of legs, antennae, and bristles in *Drosophila* (Adachi-Yamada et al. 2005). In this regard, cleft palate and hydronephrosis, two of the teratogenic end points of dioxin exposure during mouse embryonic development (Abbott et al. 1994), might be the result of the derailment of endogenous AHR morphogenetic functions. This might also be the case for the cardiac toxicity of TCDD (Aragon et al. 2008) and the recently found crosstalk between AHR and WNT in tissue regeneration in zebrafish (Mathew et al. 2008). Although the orthodox view is that AHR activation is followed by changes in its compartmentalization within the cell, little is known about the role that endogenous AHR ligands, such as tryptophan photoproducts (Wei et al. 1999), lipoxin A4 (Schaldach et al. 1999), cAMP (Oesch-Bartlomowicz et al. 2005), or indole derivatives (Song et al. 2002), might have in the nuclear translocation of AHR in the absence of xenobiotic ligands. Growing evidence indicates that the AHR may be in a continued state of nucleocytoplasmic shuttling (Pollenz 2002; Song and Pollenz 2002), which may provide the necessary temporal localization to bind to chromatin and exert its regulatory functions. It would be reasonable to conclude that the AHR possesses a set of endogenous functions that are independent of its activation as a transcription factor by xenobiotic ligands and that these functions may perform a regulatory role during development.

Exposure to either of the two xenobiotic ligands in this study, BaP or TCDD, causes a pronounced shift in the gene clusters targeted by the AHR, such that many of those identified in naive cells were no longer recognized by the receptor when activated by either BaP or TCDD, whereas the metabolism of xenobiotics pathway in KEGG emerged as a targeted pathway in treated cells. It was apparent that each ligand activated the AHR to target gene clusters in common with those in naive cells as well as new clusters, some common to receptors activated by both ligands, and some ligand specific. For example, most clusters in group C (Figure 5), involved in vascular development, positive regulation of transcription, and metabolic processes, were targeted in naive cells and to a lesser extent in cells treated with either ligand, whereas clusters of genes involved in regulation of growth and transcription from RNA polymerase II promoters (group A) and in nervous system development, embryonic development, eye development, tube morphogenesis, angiogenesis, and patterning of blood vessels (group E) were AHR targets only in naive cells. These findings may have serious implications on the etiology of diseases caused by AHR ligands, because any observed changes in gene regulation resulting from exposure might be due not to new regulatory units being recognized by the activated receptor, but to loss of regulation of preexisting ones in the unexposed organism. Given the known role of AHR ligands and other environmental agents in cardiovascular disease, it seems reasonable to conclude that the etiology of environmental cardiovascular disease may be connected with the AHR clusters involved in cardiovascular function, angiogenesis, or patterning of blood vessels (groups C and E). Similarly, the etiology of proliferative diseases may depend on gene clusters regulating cell death or proliferation and mitosis (groups D, F, and G). A case in point in this context may be the tumor suppressor gene *Pten*, which we verified by QPCR as being more highly bound by AHR in naive cells than in BaP- or TCDD-treated cells. Ablation of the *Bmpr1a* gene in the hair follicle bulge has recently been shown to signal PTEN inhibition, induction of follicle stem cell proliferation and inhibition of differentiation through β-catenin stabilization and LEF1 activation (Kobielak et al. 2007). PTEN inhibition by TCDD exposure might initiate a similar sequence of events leading to chloracne, the hyperproliferative disease of the hair follicle that is a consequence of TCDD-induced disease in humans.

An extended group of gene clusters (groups E and H) is involved in biological processes governing embryonic development and patterning of developmental structures. Several genes in these clusters are homeobox genes, which in turn control the concerted cascade of expression of other genes, potentially amplifying the regulatory capabilities of the AHR. Exposure to AHR ligands during embryonic life may derail the concerted expression of developmental genes and, in addition, alter the normal patterns of epigenetic modifications of these genes, an effect that might persist throughout the lifetime of the organism and possibly be a determinant of disease susceptibility in the adult.

The results from the present study provide a unique resource that should advance our ability to establish needed gene–environment connections between environmental disease and disturbances of gene regulation patterns at the level of the whole genome. We should emphasize that it is evident that some of the networks uncovered here will be limited to hepatoma cells in culture and include genes bound by a poised receptor but not necessarily transcriptionally activated. In an organism, each cell lineage or tissue will have its own specific target network, including unique as well as overlapping domains. Given the large enrichment of developmental clusters in the AHR target network, a higher order understanding of the connections among disease state, environment, and AHR target genes will require extending these studies to the diverse tissues of the developing organism and defining a target network map at the level of the whole organism, a task that will be feasible after the knowledge base developed from the work presented here. Because AHR activation by its ligands generates chromatin modifications and changes in histone code (Schnekenburger et al. 2007), it is likely that any environmental injury that might occur in the developing embryo as a consequence of exposure to AHR ligands will cause persistent changes of gene expression for the life of the organism. The molecular basis of adult environmental disease might be rooted in exposures occurring during fetal life; if this were the case, understanding adult environmental diseases may require the synergistic interaction of toxicology and developmental biology.

## References

[b1-ehp-117-1139] Abbott BD, Perdew GH, Birnbaum LS (1994). Ah receptor in embryonic mouse palate and effects of TCDD on receptor expression. Toxicol Appl Pharmacol.

[b2-ehp-117-1139] Adachi-Yamada T, Harumoto T, Sakurai K, Ueda R, Saigo K, O’Connor MB (2005). Wing-to-leg homeosis by spineless causes apoptosis regulated by Fish-lips, a novel leucine-rich repeat transmembrane protein. Mol Cell Biol.

[b3-ehp-117-1139] Aragon AC, Kopf PG, Campen MJ, Huwe JK, Walker MK (2008). In utero and lactational 2,3,7,8-tetrachlorodibenzo-*p*-dioxin exposure: effects on fetal and adult cardiac gene expression and adult cardiac and renal morphology. Toxicol Sci.

[b4-ehp-117-1139] Barouki R, Coumoul X, Fernandez-Salguero PM (2007). The aryl hydrocarbon receptor, more than a xenobiotic-interacting protein. FEBS Lett.

[b5-ehp-117-1139] Benjamini Y, Hochberg Y (1995). Controlling the false discovery rate: a practical and powerful approach to multiple testing. J R Stat Soc Series B Stat Methodol.

[b6-ehp-117-1139] Birnbaum LS, Harris MW, Stocking LM, Clark AM, Morrissey RE (1989). Retinoic acid and 2,3,7,8-tetrachlorodibenzo-*p*-dioxin selectively enhance teratogenesis in C57BL/6N mice. Toxicol Appl Pharmacol.

[b7-ehp-117-1139] Bock KW, Kohle C (2006). Ah receptor: dioxin-mediated toxic responses as hints to deregulated physiologic functions. Biochem Pharmacol.

[b8-ehp-117-1139] Chang X, Fan Y, Karyala S, Schwemberger S, Tomlinson CR, Sartor MA (2007). Ligand-independent regulation of transforming growth factor beta1 expression and cell cycle progression by the aryl hydrocarbon receptor. Mol Cell Biol.

[b9-ehp-117-1139] Crews ST, Fan CM (1999). Remembrance of things PAS: regulation of development by bHLH-PAS proteins. Curr Opin Genet Dev.

[b10-ehp-117-1139] Efron B (2004). Large-scale simultaneous hypothesis testing: the choice of a null hypothesis. J Am Stat Assoc.

[b11-ehp-117-1139] Fernandez-Salguero P, Pineau T, Hilbert DM, McPhail T, Lee SS, Kimura S (1995). Immune system impairment and hepatic fibrosis in mice lacking the dioxin-binding Ah receptor. Science.

[b12-ehp-117-1139] Fernandez-Salguero PM, Ward JM, Sundberg JP, Gonzalez FJ (1997). Lesions of aryl-hydrocarbon receptor-deficient mice. Vet Pathol.

[b13-ehp-117-1139] Frericks M, Meissner M, Esser C (2007). Microarray analysis of the AHR system: tissue-specific flexibility in signal and target genes. Toxicol Appl Pharmacol.

[b14-ehp-117-1139] Ge N-L, Elferink CJ (1998). A direct interaction between the aryl hydrocarbon receptor and retinoblatoma protein. J Biol Chem.

[b15-ehp-117-1139] Gene Ontology (2009). Gene Ontology Home Page.

[b16-ehp-117-1139] Hahn ME (2002). Aryl hydrocarbon receptors: diversity and evolution. Chem Biol Interact.

[b17-ehp-117-1139] Hankinson O (1995). The aryl hydrocarbon receptor complex. Annu Rev Pharmacol Toxicol.

[b18-ehp-117-1139] Hestermann EV, Brown M (2003). Agonist and chemopreventative ligands induce differential transcriptional cofactor recruitment by aryl hydrocarbon receptor. Mol Cell Biol.

[b19-ehp-117-1139] Hoffer A, Chang C-Y, Puga A (1996). Dioxin induces *fos* and *jun* gene expression by Ah receptor dependent- and independent- pathways. Toxicol Appl Pharmacol.

[b20-ehp-117-1139] Klinge CM, Kaur K, Swanson HI (2000). The aryl hydrocarbon receptor interacts with estrogen receptor alpha and orphan receptors COUP-TFI and ERRalpha1. Arch Biochem Biophys.

[b21-ehp-117-1139] Kobielak K, Stokes N, de la Cruz J, Polak L, Fuchs E (2007). Loss of a quiescent niche but not follicle stem cells in the absence of bone morphogenetic protein signaling. Proc Natl Acad Sci U S A.

[b22-ehp-117-1139] Kyoto Encyclopedia (2009). Kyoto Encyclopedia of Genes and Genomes.

[b23-ehp-117-1139] Lahvis GP, Lindell SL, Thomas RS, McCuskey RS, Murphy C, Glover E (2000). Portosystemic shunting and persistent fetal vascular structures in aryl hydrocarbon receptor-deficient mice. Proc Natl Acad Sci U S A.

[b24-ehp-117-1139] Lahvis GP, Pyzalski RW, Glover E, Pitot HC, McElwee MK, Bradfield CA (2005). The aryl hydrocarbon receptor is required for developmental closure of the ductus venosus in the neonatal mouse. Mol Pharmacol.

[b25-ehp-117-1139] Marlowe JL, Fan Y, Chang X, Peng L, Knudsen ES, Xia Y (2008). The Ah receptor binds to E2F1 and inhibits E2F1-induced apoptosis. Mol Biol Cell.

[b26-ehp-117-1139] Marlowe JL, Puga A (2005). Aryl hydrocarbon receptor, cell cycle regulation, toxicity, and tumorigenesis. J Cell Biochem.

[b27-ehp-117-1139] Mathew LK, Sengupta SS, Ladu J, Andreasen EA, Tanguay RL (2008). Crosstalk between AHR and Wnt signaling through R-Spondin1 impairs tissue regeneration in zebrafish. FASEB J.

[b28-ehp-117-1139] Matys V, Fricke E, Geffers R, Gossling E, Haubrock M, Hehl R (2003). TRANSFAC: transcriptional regulation, from patterns to profiles. Nucleic Acids Res.

[b29-ehp-117-1139] Oesch-Bartlomowicz B, Huelster A, Wiss O, Antoniou-Lipfert P, Dietrich C, Arand M (2005). Aryl hydrocarbon receptor activation by cAMP vs. dioxin: divergent signaling pathways. Proc Natl Acad Sci U S A.

[b30-ehp-117-1139] Pocar P, Fischer B, Klonisch T, Hombach-Klonisch S (2005). Molecular interactions of the aryl hydrocarbon receptor and its biological and toxicological relevance for reproduction. Reproduction.

[b31-ehp-117-1139] Pollenz RS (2002). The mechanism of AH receptor protein down-regulation (degradation) and its impact on AH receptor-mediated gene regulation. Chem Biol Interact.

[b32-ehp-117-1139] Qin H, Powell-Coffman JA (2004). The Caenorhabditis elegans aryl hydrocarbon receptor, AHR-1, regulates neuronal development. Dev Biol.

[b33-ehp-117-1139] R Project for Statistical Computing (2009). http://www.r-project.org/.

[b34-ehp-117-1139] Sartor MA, Tomlinson CR, Wesselkamper SC, Sivaganesan S, Leikauf GD, Medvedovic M (2006). Intensity-based hierarchical Bayes method improves testing for differentially expressed genes in microarray experiments. BMC Bioinformatics.

[b35-ehp-117-1139] Schaldach CM, Riby J, Bjeldanes LF (1999). Lipoxin A4: a new class of ligand for the Ah receptor. Biochemistry.

[b36-ehp-117-1139] Schnekenburger M, Peng L, Puga A (2007). HDAC1 bound to the Cyp1a1 promoter blocks histone acetylation associated with Ah receptor-mediated trans-activation. Biochim Biophys Acta.

[b37-ehp-117-1139] Smith AD, Sumazin P, Zhang MQ (2005). Identifying tissue-selective transcription factor binding sites in vertebrate promoters. Proc Natl Acad Sci U S A.

[b38-ehp-117-1139] Smyth GK (2004). Linear models and empirical Bayes methods for assessing differential expression in microarray experiments. Stat Appl Genet Mol Biol.

[b39-ehp-117-1139] Song J, Clagett-Dame M, Peterson RE, Hahn ME, Westler WM, Sicinski RR (2002). A ligand for the aryl hydrocarbon receptor isolated from lung. Proc Natl Acad Sci U S A.

[b40-ehp-117-1139] Song Z, Pollenz RS (2002). Ligand-dependent and independent modulation of aryl hydrocarbon receptor localization, degradation, and gene regulation. Mol Pharmacol.

[b41-ehp-117-1139] Sun W, Zhang J, Hankinson O (1997). A mutation in the aryl hydrocarbon receptor (AHR) in a cultured mammalian cell line identifies a novel region of AHR that affects DNA binding. J Biol Chem.

[b42-ehp-117-1139] Taylor J, Tibshirani R, Efron B (2005). The “miss rate” for the analysis of gene expression data. Biostatistics.

[b43-ehp-117-1139] Wei YD, Rannug U, Rannug A (1999). UV-induced CYP1A1 gene expression in human cells is mediated by tryptophan. Chem Biol Interact.

